# Deep Reinforcement Learning for Traffic Signal Control Model and Adaptation Study

**DOI:** 10.3390/s22228732

**Published:** 2022-11-11

**Authors:** Jiyuan Tan, Qian Yuan, Weiwei Guo, Na Xie, Fuyu Liu, Jing Wei, Xinwei Zhang

**Affiliations:** 1 School of Electrical and Control Engineering, North China University of Technology, Beijing 100144, China; 2 School of Management Science and Engineering, Central University of Finance and Economics, Beijing 100081, China

**Keywords:** deep reinforcement learning, sensor technology, signal optimization, state space

## Abstract

Deep reinforcement learning provides a new approach to solving complex signal optimization problems at intersections. Earlier studies were limited to traditional traffic detection techniques, and the obtained traffic information was not accurate. With the advanced in technology, we can obtain highly accurate information on the traffic states by advanced detector technology. This provides an accurate source of data for deep reinforcement learning. There are many intersections in the urban network. To successfully apply deep reinforcement learning in a situation closer to reality, we need to consider the problem of extending the knowledge gained from the training to new scenarios. This study used advanced sensor technology as a data source to explore the variation pattern of state space under different traffic scenarios. It analyzes the relationship between the traffic demand and the actual traffic states. The model learned more from a more comprehensive state space of traffic. This model was successful applied to new traffic scenarios without additional training. Compared our proposed model with the popular SAC signal control model, the result shows that the average delay of the DQN model is 5.13 s and the SAC model is 6.52 s. Therefore, our model exhibits better control performance.

## 1. Introduction

Due to the limited space on urban roads, a series of traffic problems, including traffic congestion and traffic accidents, have arisen. This causes serious economic losses and constrains the sustainable development of cities. Increasing traffic congestion has become a common problem in cities. To address this problem, some researchers have proposed measures to build an intelligent traffic system using intelligent technologies. Traffic signal control is the core element of an intelligent traffic system and an important means to solve traffic problems [1].

With the development of communication technology, sensor technology in urban traffic systems has been enhanced to efficiently and accurately acquire complex traffic information to improve traffic signal control strategies [2]. Previous researchers have utilized a loop coil sensor to collect traffic flow data. The traffic data are utilized as the basis for setting the parameters of the traffic signal to achieve fixed timing optimization [3]. Traditional loop coil sensor technology can detect the number of vehicles but cannot identify the vehicle type and continuous traffic flow [4]. Manual surveys are often required to determine the distribution of vehicle types. The signal control strategies implemented with such traffic data are not accurate.

To obtain traffic data conveniently, video and radar sensor technology have been widely applied, which detects a larger range than the loop coil sensor and obtains traffic information for a cross-section. The video sensor captures a real-time scene of the intersection by camera. It is passed to the handler for processing to achieve traffic count and speed recognition [5]. Previous researchers have performed large-scale traffic flow data collection and analysis based on video sensors, overcoming the limitations of image processing, which provides support for traffic signal control [6]. The radar sensor enables real-time detection of multiple lanes of traffic, detecting data such as traffic flow, speed, and occupancy, as well as providing real-time traffic condition information [7].

The Internet of Vehicles achieves the network connection of vehicle to cloud platform, vehicle to vehicle, vehicle to road, and vehicle to people through information and communication technologies [8]. At present, relying on communication technology, connected vehicles can also be used as sensors to collect high-precision status information, which can lay the foundation for the realization of real-time, efficient, and accurate traffic control [9,10]. In summary, a comparison of the sensor technologies is shown in Table 1 and Table 2.

In past decades, researchers relied on loop coil sensors to obtain traffic status information. Fixed-timing signal control methods were utilized to improve traffic efficiency and capacity. Subsequently, adaptive control methods have been widely applied in signal control systems. Compared with fixed timing methods, these improved the flexibility of signal control and vehicle throughput efficiency [11]. Since these traditional signal control methods optimize signals across time periods and cycles [12], it is difficult to deal with the complex time-varying traffic demand. Therefore, some researchers have proposed data-driven models [13,14]. However, traffic information at intersections is complex and diverse. Limited by the sensor technology, the effect of data-driven models with low-precision traffic status information is not ideal. With the development of technologies such as vehicle networking, the traffic information data that can be obtained are more refined. This enables researchers to develop new signal control models. Among these, the application of deep reinforcement learning (DRL) to the field of traffic signal control has become a research hotspot.

Much research has been conducted to solve traffic signal control problems with the DRL algorithm based on advanced sensor technology, which has achieved great effects in practice. However, cities have many intersections and a high dimensionality of traffic demand. Each intersection needs to be trained with a corresponding set of signal control models [15]. Retraining the model to calibrate the parameters takes a significant amount of time [16,17,18]. Hence, model training is a problem.

Therefore, several important issues remain to be solved, which include:

(1) Finding a suitable model that can be applied to more than one intersection scenario;

(2) Determining the relationship between the traffic demand at the intersection and the actual traffic state;

(3) Designing a traffic scenario that represents a more comprehensive traffic demand.

In this paper, we research an adaptable DRL signal control model to deal with these problems.

## 2. Related Work

The DRL algorithm is a combination of reinforcement learning and deep learning. Reinforcement learning acquires knowledge by autonomously interacting with the environment through a trial-and-error learning model, which is similar to a human being [19]. Minh et al. first proposed the deep Q-network model [20], which is suitable for processing high-dimensional data. Some researchers have applied deep reinforcement learning (DRL) to the field of signal control and achieved positive effects in solving complex traffic congestion problems.

Much of the existing work uses deep reinforcement learning techniques to solve complex signal optimization problems at intersections. Firstly, DRL traffic signal control measures can be divided into three kinds: value-based, policy-based, and actor–critic approaches, which are shown in Table 3. According to the training policy, the DRL signal control methods can be divided into on-policy and off-policy. The deep Q-learning (DQN) is an on-policy algorithm. The off-policy signal control model does not interact with the environment in real-time. It takes a batch of samples from the experience replay buffer for learning [21,22,23]. Kim proposed a cooperative traffic signal control with traffic flow prediction (TFP-CTSC) for a multi-intersection. The results indicated that the model improved the travel efficiency of vehicles on the road network [24]. Rasheed proposed a multiagent DQN (MADQN) and investigated its use to further address the problem of dimensionality under traffic network scenarios with high traffic volume and disturbances. The simulation results showed that the proposed scheme reduced the total travel time of the vehicles [25]. Song transferred the well-trained action policy of a previous DQN model into a target model under similar traffic scenarios. The results indicated that, compared to the directly trained DQN, transfer-based models could improve both the training efficiency and model performance [18].

The on-policy signal control model interacts with the environment in real-time to obtain an optimized policy, which can optimize while learning [26]. The PG is an on-policy algorithm, and it learns a parameterized strategy function with sampled episode return [27]. Rizzo et al. proposed the time critic PG technique to avoid jams in heavy traffic volumes. Policy-based algorithms are effective in high-dimensional and continuous action spaces [28].

The SAC algorithm is an off-policy actor–critic approach that has excellent sampling efficiency [29]. The off-policy approach solves the problems of difficult data collection and the high cost and risky implementation process in an on-policy approach. It is of great importance in practical applications. Mao evaluated seven prevailing DRL algorithms from two aspects: training and execution performance. The testing results indicated that the soft actor–critic (SAC) outperformed other DRL algorithms and the maximum pressure method in most cases [30]. Chu presented the advantage actor–critic (A2C) to stabilize the learning procedure, and the results demonstrated its optimality, robustness, and sample efficiency over other decentralized MARL algorithms [15]. Li proposed a PPO algorithm to optimize the fairness of all drivers’ waiting times, and the results showed the algorithm efficiently optimized the fairness criterion and had a more stable performance than the A2C model [31].

These researchers enhanced the performance of the DRL method by different means. The model usually performed better in the same or similar traffic scenarios. However, it cannot be adapted to new traffic scenarios. It takes time and effort to train a specific DQN model for each intersection in reality [18]. Therefore, the question of how to find a signal control model that can be adapted to new traffic scenarios needs to be addressed. In this research, we search for a method that can train adaptive models based on the DRL approach. First and foremost, a suitable DRL algorithm needs to be selected as the testing algorithm. Mao et al. showed that the value-based/off-policy optimization approach had good sampling efficiency. It showed better performance than other approaches for a discrete action space decision-making problem, such as traffic signal control [30]. Due to the strong correlation between traffic states, the algorithm training needs to maintain the independence between samples. In addition, the corresponding road facilities are not yet available, and there are technical difficulties in the implementation of an on-policy strategy. Therefore, off-policy was selected to optimize the signal control model in this paper. The focus of the research was not to optimize the performance of the algorithm itself but to explore how to train a DRL signal control model that could be generalized to new traffic scenarios with an adaptable performance. We examined several value-based/off-policy algorithms with similar effects. Among them, the DQN algorithm is a mature form of urban signal control [32,33]. We used this method as the test algorithm for this research.

**Figure 1:**
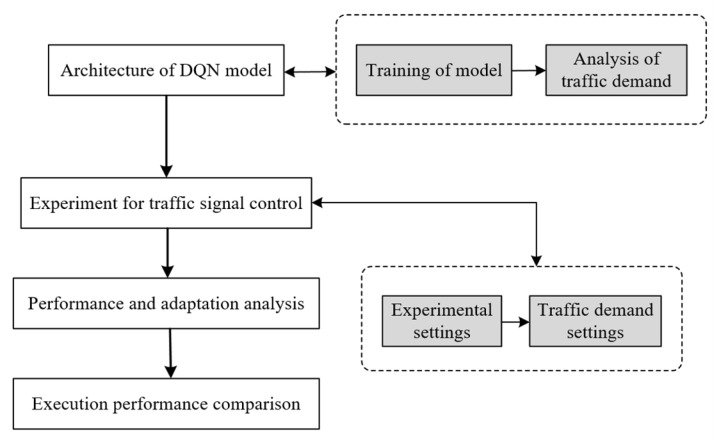
Overall framework of the research.

In the following section, we describe the construction of a traffic simulation platform based on the DQN algorithm to simulate the signal control. From the perspective of enriching the sample, a comprehensive traffic demand was designed so that the model could “see” as many traffic states as possible to improve the model’s adaptability to new traffic scenarios. The relationship between the input traffic demand and the actual traffic flow state was analyzed to aid in designing efficient traffic demand. The feasibility and effectiveness of the model proposed in the research were verified by comparing it with other signal control methods. Ultimately, we obtained a model that could be adapted to new traffic scenarios, which saves the time and effort consumed by repeated training at different intersections. The overall framework of the research is shown in Figure 1.

## 3. Materials and Methods

In this section, we first describe the framework of the DQN traffic signal model, including the overall architecture of the DQN algorithm, the definition of the reward function, and the setting of the observation state and action. Secondly, we explain the training process and method of the model. Finally, we analyze the relationship between traffic demand and traffic states.

### 3.1. Architecture of the DQN Model

**Figure 2:**
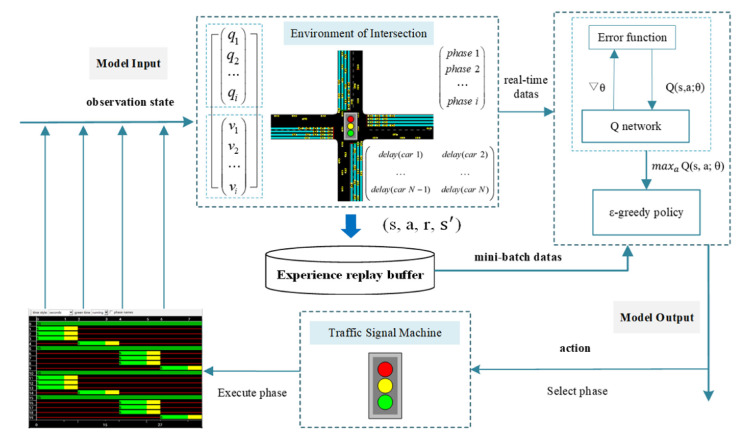
The overall architecture of the DQN model.

The traffic signal control problem is described as an optimization problem with deep reinforcement learning. The overall framework of the DQN signal control model is shown in Figure 2. First, we observed the environment to obtain the traffic status of the intersection, such as vehicle speed, queue length, etc. The real-time traffic status information was input into the Q network, and the Q value corresponding to each signal decision action was the output. According to the action selection strategy, the signal decision action with the maximum Q value was selected. It was sent to the signal machine for execution. Then, feedback was provided to measure the traffic operation of the intersection. The process was repeated until the objective of maximizing the cumulative reward $R_{t}=\sum_{n=0}^{\infty }\gamma^{n}r_{t+n}$ was reached. The traffic parameters, such as the traffic state information, signal decision action, reward value, and the traffic state at the next time, were stored in the quadratic form in the experience replay buffer. The Q network was trained with a batch of samples until convergence, and the optimal mapping of the “state-action” was obtained.

It is necessary to determine the traffic parameter indicators of the model, such as the traffic state information, the signal actions, and the reward functions for evaluating the control effect. The model observes the intersection environment at time t to obtain the information of the traffic state $s_{t}$. The queue length was chosen to characterize the state of the intersection, which describes the distribution of the vehicle queue lengths on each lane, $s_{t}\in S$. The model chose a corresponding action $a_{t}$, which is defined as the selection of the signal phase according to the traffic state, $a_{t}\in A$. The environment provided feedback on the action taken by the signal at the next moment. The reward function $r\left(s_{t},a_{t}\right)$ is defined as the inverse of the average vehicle cumulative delay time, which is used to quantify the impact of the action.

#### 3.1.1. Reward Function

The reward value reflects the impact of the model after deciding. The reward function influences the final performance of the model, which includes vehicle delay time, queue length, waiting time and vehicle speed, etc. The objective of the study is to improve the efficiency of vehicle movement and reduce the delay time of vehicles at the intersection. Therefore, the opposite of the average vehicle delay time is set as the reward function, and its expression is

(1) $$r\left(s_{t},a_{t}\right)=-\sum_{\tau =t}^{t+\Delta t}\alpha \cdot d_{t}^{l}$$

where α is the weighting factor; $d_{t}^{l}$ is the average delay time for each lane at the time t, $l_{i}\in L$.

#### 3.1.2. Observation State

Traffic observation states are the critical factors for signals to make decisions, and each observation state can contain one or more substates, $s_{t}=\left({s_{t}}^{1},{s_{t}}^{2},{s_{t}}^{3},...,{s_{t}}^{j}\right)$. Researchers usually select traffic information such as the vehicle location, the average speed, the traffic throughput, the queue length, and the average waiting time as the observed states. The corresponding observation matrix was constructed as the input of the DQN algorithm.

**Figure 3:**
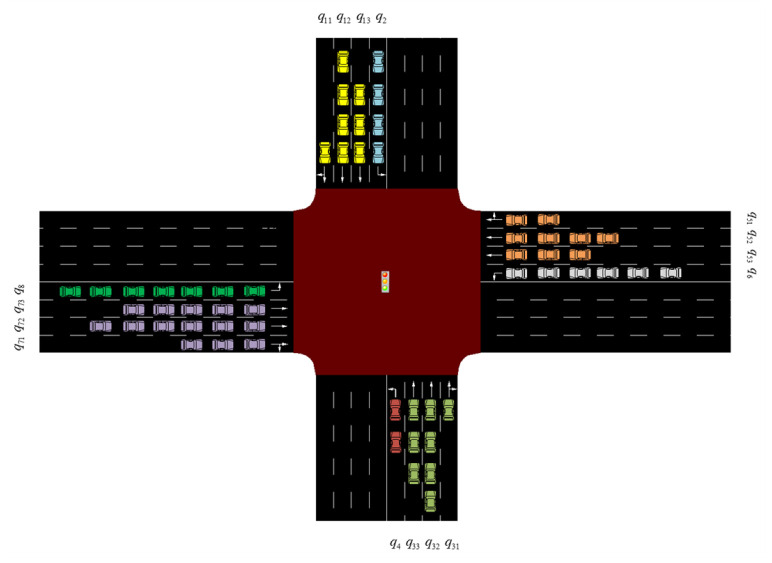
Diagram of the observation state.

The queue length is selected as the observed traffic state. The queue length indicates the number of vehicles waiting in the queue on the lane, which changes with the arrival and departure of vehicles. The queue length $q_{i}$ of each lane $l_{i}$ at the intersection is collected. As shown in Figure 3, the traffic flow at the intersection is divided into eight flow directions. The traffic observation state is an eight-dimensional matrix:

(2) $$s_{t}=\left[q_{1},q_{2},q_{3},q_{4},q_{5},q_{6},q_{7},q_{8}\right]$$

Among these, $q_{1}=\max\left\{q_{11},q_{12},q_{13}\right\}$, $q_{3}=\max\left\{q_{31},q_{32},q_{33}\right\}$, $q_{5}=\max\left\{q_{51},q_{52},q_{53}\right\}$, and $q_{7}=\max\left\{q_{71},q_{72},q_{73}\right\}$.

#### 3.1.3. Action Settings

Signals at intersections make appropriate decisions based on the current traffic situation. The decision variables for signal control include the phase green time, the phase switching, and the phase selection. The phase green time optimizes the signal by adjusting the green duration. The phase switching is based on a predefined phase sequence that determines the duration of the green light and switches the signal to the next preset phase. Phase switching is the process of deciding whether to switch the signal to the next phase in a predefined phase sequence. Phase selection is more flexible than phase switching in that it selects the phase to be performed from a set containing multiple phases.

**Figure 4:**
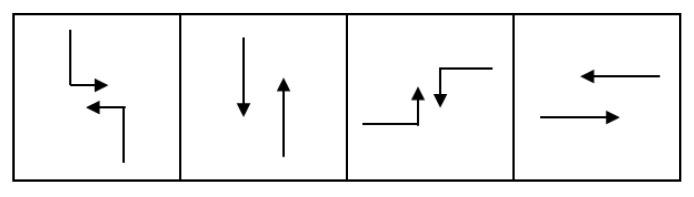
The set of phases.There are four possible phases.

Phase selection is set as the possible actions of the model, which decide the next phase according to the traffic states [34]. Firstly, four feasible phases are defined for the signal system, as shown in Figure 4. The set of phases is phase={NSL,NSS,WEL,WES}.

**Figure 5:**
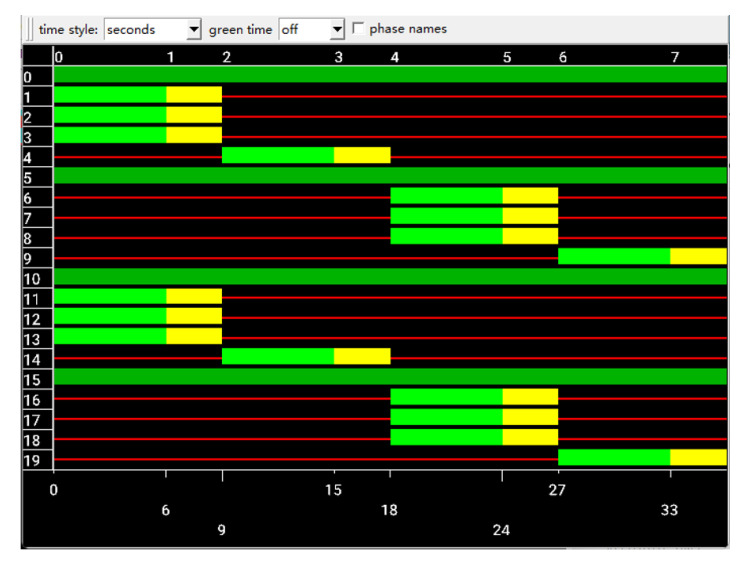
Phase selection.The diagram is derived from the interface of SUMO simulation.

The set of phase selections is Action={0,1,2,3}. The phase to be executed is selected according to the action of the Q network output. The phase sequence is shown in Figure 5. The specific decision process proceeds as follows: when $a_{t}=0$, execute the phase 0. When $a_{t}=1$, execute the phase 2. When $a_{t}=2$, execute the phase 4. When $a_{t}=3$, execute the phase 6.

### 3.2. Training of the DQN Model

The SUMO and Python software are utilized to construct a traffic simulation platform for the experiments. The main functions of SUMO include building road networks, generating traffic demands, and obtaining various traffic evaluation indicators. The function of Python is to implement the DQN algorithm and interact with SUMO in real-time.

We set up two main files to run the SUMO simulation, which include the following:

(1) Road network file (net.xml): We built the road network and set up the road details in this file.

(2) Traffic routing file (rou.xml): We input the traffic requirements and generated the traffic scenarios in this file.

There are other files, which include vehicle description files and detector description files, which could be added to run a superior simulation.

We designed the DQN algorithm and determined the hyperparameters. When setting the traffic simulation time, we considered that after generating the traffic demand in the road network, the input traffic flow needs a certain time to enter the steady state to ensure the desired duration of traffic demand stability and improve the possibility of the model to explore the knowledge fully. Therefore, the simulation duration must be sufficient. The number of simulations needed to ensure the convergence of the neural network. The recommended value for the discount factor was 0.9, the recommended value for the batch size was 400, the recommended value for the learning rate was $3\times 10^{-4}$, and the recommended value for the number of neural network iterations at each sampling was 4 [15,29,35].

After initializing the parameters, Python interacted with SUMO in real time to obtain real-time traffic status information of the intersection. The traffic status was fed into the neural network. Then, the neural network output the Q values corresponding to each phase. We selected the phase to be executed by ε−greedy and sent it down to the signal for execution. The average delay of the current moment was obtained to evaluate the control effect of the current phase. The traffic state, the selected phase, the average delay, and the traffic state at the next time step were stored in the experience replay buffer. Finally, a batch of samples was randomly drawn from the experience pool and used to update the weights of the neural network.

When the input layer of the neural network is $s_{t}$, the output will be $Q^{\pi }\left(s_{t}\right)$. When the input layer of the neural network is $s_{t+1}$, the output will be $Q^{\pi }\left(s_{t+1}\right)$. The goal of updating the weights of the neural network was to obtain the difference between $Q^{\pi }\left(s_{t}\right)$ and $Q^{\pi }\left(s_{t+1}\right)$ closer to $r_{t}$. The neural network output the actual *Q*-value, while the target value was approximated using the value corresponding to the action with the largest *Q*-value in the next state. The equation for updating is:

(3) $$Q_{t}\left(s_{t},a_{t}\right)=Q_{t}\left(s_{t},a_{t}\right)+\alpha \left[r\left(s_{t},a_{t}\right)+\gamma \max_{a}Q\left(s_{t+1},a\right)-Q_{t}\left(s_{t},a_{t}\right)\right]$$

where $r\left(s_{t},a_{t}\right)$ is the reward. α,γ is the discount factor.

In addition, we designed a suitable traffic scenario to test the trained model. The model testing was divided into the same demand scenario and a new demand scenario. The same demand scenario testing referred to the testing under the same test scenario and training scenario, which was used to verify the performance of the completed training model. The new demand scenario testing referred to testing under different test scenarios from the training scenarios and was used to verify the adaptive performance of the model. Finally, the DQN model of the research was analyzed and compared to several existing traffic signal control methods.

### 3.3. The Relationship between the Traffic Demand and Traffic States

To construct traffic states with various degrees of equilibrium, we designed n traffic scenarios. In each traffic scenario, different percentages of traffic flow were assigned to multiple directions of the intersection. The designed traffic scenarios represented several typical traffic flow states, extreme traffic states, perfectly balanced traffic states, perfectly balanced and mildly unbalanced traffic states, and fully balanced traffic states.

Exploring empirical knowledge plays a key role in deep reinforcement learning. The generalized application of the model can be limited by restricted empirical knowledge. To obtain models with adaptability, it is necessary to provide rich empirical knowledge for model exploration. Therefore, the study classified the traffic demand by the balance level of traffic distribution. Each model was equipped with traffic demand scenarios with different levels of balance.

There are *n* periods to input traffic, and the total number of vehicles at period *t* is $Q_{t}$. Assume that the simulation time for each period is *s* and the model is simulated once in time T=n·s. To distinguish between traffic states with different levels of balance. The intersection has *m* directions and assigns traffic flow in direction *i* is $q_{i}\left(t\right)$, $\sum_{i=1}^{m}q_{i}\left(t\right)=Q_{t}$. Therefore, the percentage of the assigned traffic flow in direction *i* is $r_{i}\left(t\right)$, $r_{i}\left(t\right)=\frac{q_{i}\left(t\right)}{Q_{i}\left(t\right)}$.

Traffic scenarios set up traffic arrivals by time series. However, the actual traffic state is influenced by the signal control and has a strong time correlation. Therefore, to study how to set a more comprehensive and effective demand scenario, the actual traffic state is to be analyzed.Since the traffic parameter of queue length is a continuous value, the dimensionality of its solution is large. In addition, the traffic states with similar values of queue length are similar, and there is no abrupt variation in traffic states. Therefore, we decided to discretize the queue length of intersections to improve the efficiency of the traffic state analysis and reduce the computational consumption. The specific discretization process: we divided the intersection into *x* flow directions and divided the queue length into *k* segments. The unit queue length interval of the *i*-th segment is $l_{i}$, $q\in [q_{i},q_{i+1})$. The final number of state spaces for a certain flow direction is obtained as $a=\sum_{i=1}^{k}\frac{q_{i}-q_{i-1}}{l_{i}}$. Therefore, the number of state spaces at the intersection is $a^{x}$.

## 4. Results

### 4.1. Experimental Settings

The intersection is designed as a “cross-shaped” intersection. The four directions of the intersection are four lanes in both directions, including a left-turn lane, two straight lanes, and a right-turn lane. The length of the lane is 750 m. The length of the vehicle is 5 m, the maximum driving speed is 25 m/s, and the average speed is 10 m/s. In addition, detailed information about the traffic demand is described in the next subsection.

The initialized hyperparameters of the DQN model are set as shown in Table 4.

### 4.2. Traffic Demand Settings

In this section, we describe the design of four representative traffic scenarios as the input for the models. The intersection had four directions, including east, west, north, and south. The percentage of the traffic flow was $r_{1}\left(t\right),r_{2}\left(t\right),r_{3}\left(t\right),r_{4}\left(t\right)$. The traffic flow was input in five time periods. The simulation time for one period was set to 1200 s to ensure that the model was explored sufficiently. The designed traffic scenarios represented several typical traffic flow states, which were extreme traffic states, perfectly balanced traffic states, perfectly balanced and mildly unbalanced traffic states, and fully balanced traffic states.

**Figure 6:**
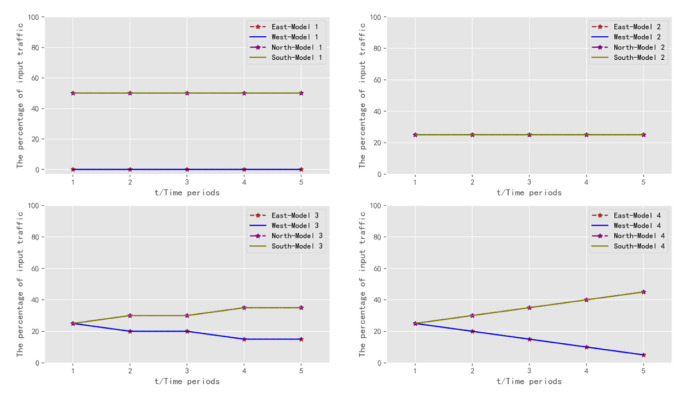
Diagram of traffic scenarios for training: allocation of vehicles in each direction.

Model 1 represented a traffic scenario containing extreme traffic conditions, where all vehicles at the intersection were assigned to the south and north directions, and no vehicles were assigned to any other direction. It had extreme unevenness in vehicle distribution. Model 2 represented a traffic scenario that contained perfectly balanced traffic states. The distribution of vehicles at the intersection was identical in each direction. Model 3 represented a traffic scenario with perfectly balanced and lightly unbalanced traffic. The distribution of vehicles at the intersection was identical for a portion of the time period. In the other part of the time period, the south and north traffic was distributed upwards a little more than in the other directions. Model 4 represented a traffic scenario with a fully balanced traffic condition. In all five time periods, the input traffic flows were the same. However, the percentage of vehicles distributed in the south and north directions gradually became larger, and the percentage of vehicles distributed in the east and west directions gradually became smaller. Therefore, the directional distribution of traffic flows containing fully balanced, mildly unbalanced, and more extreme traffic states were included. The vehicle allocation is shown in Table 5 and Figure 6.

The model is trained based on four demand scenarios. The observed states are discretized. There are eight flow directions at the intersection. The upper limit of queue length is the lane length $q_{max}=750\;m$, and the queue length of each flow direction is divided into three segments, which are shown in Table 6 and Table 7.

**Figure 7:**
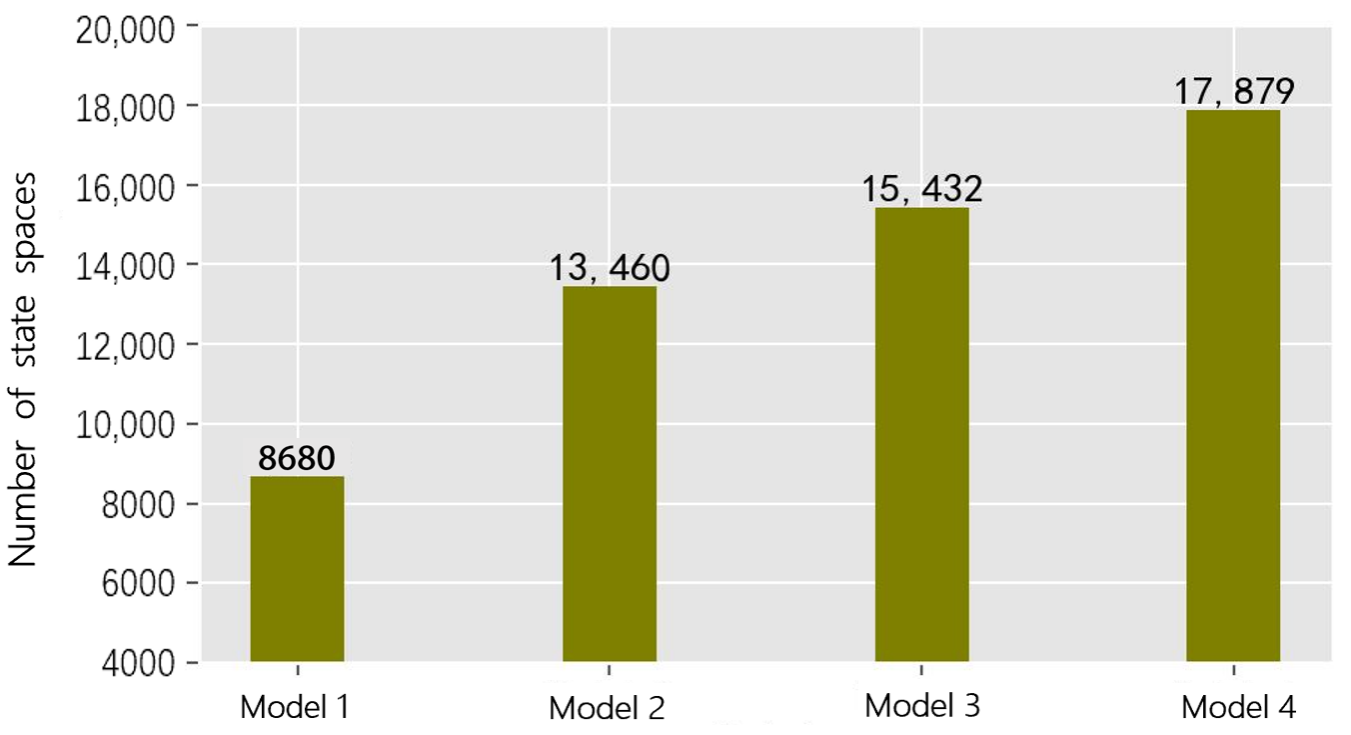
Number of state spaces for the four models.

After the data are discretized, each flow direction contains fifteen state spaces, and the number of state spaces is $15^{8}$ states at the intersection. The number of state spaces of each model is shown in Figure 7.

Model 1 represents the extreme traffic state, which contains a significantly lower number of state spaces than the other models. Models 2–4 have increasingly comprehensive traffic scenario designs, and the number of their state spaces is generally proportional. The number of state spaces does not depend entirely on the design of the traffic scenario, but is also related to the interaction between vehicles while they are moving. In addition, the temporal correlation between traffic states also affects the distribution of the state space. Therefore, we should not only design more comprehensive traffic scenarios, but also pay attention to the coverage of the actual traffic state.

**Figure 8:**
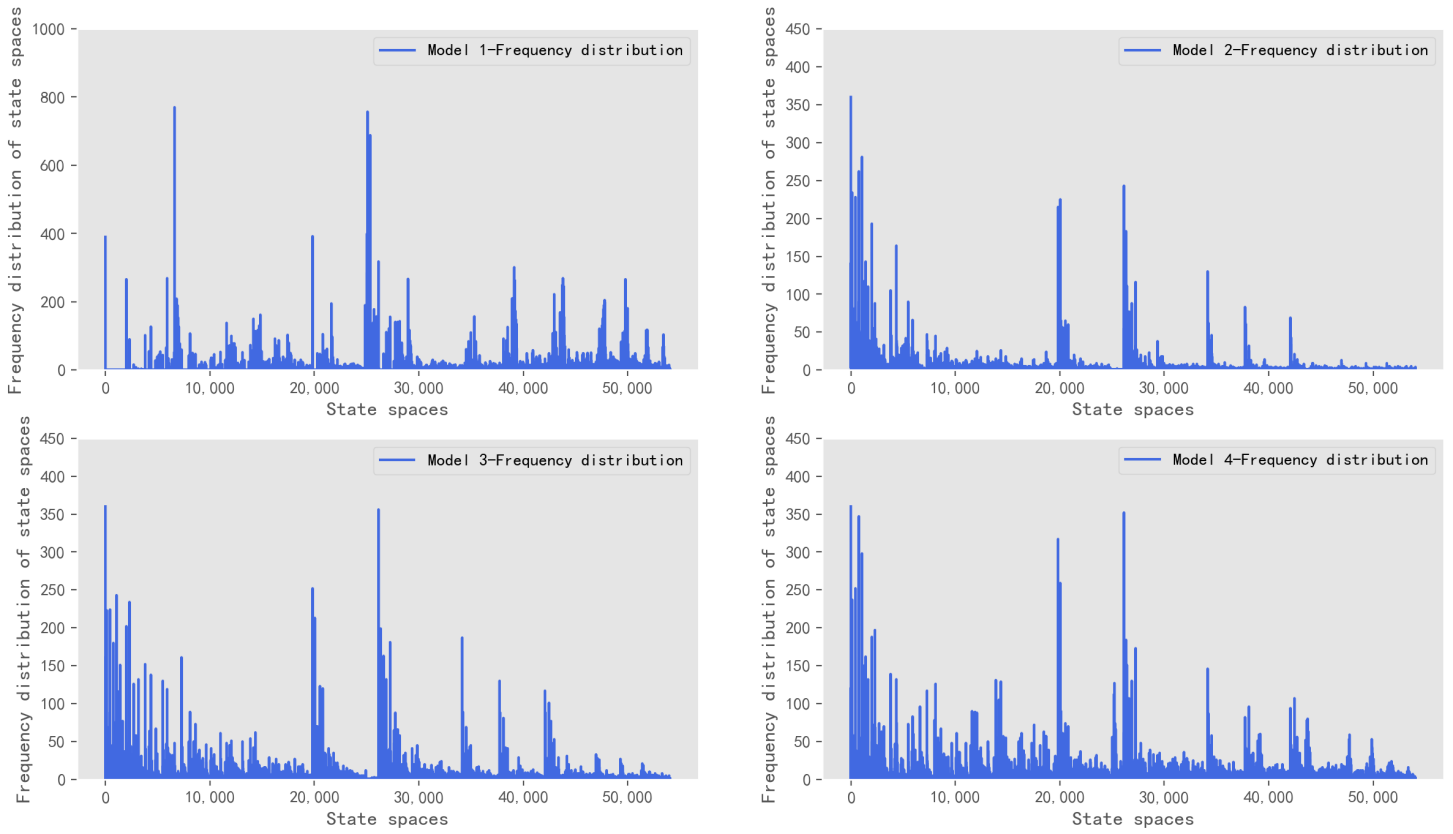
Frequency distribution of state spaces of the four models.

To further analyze the distribution of the state space, the frequency distribution of state spaces of the four models was counted, which is shown in Figure 8.

**Figure 9:**
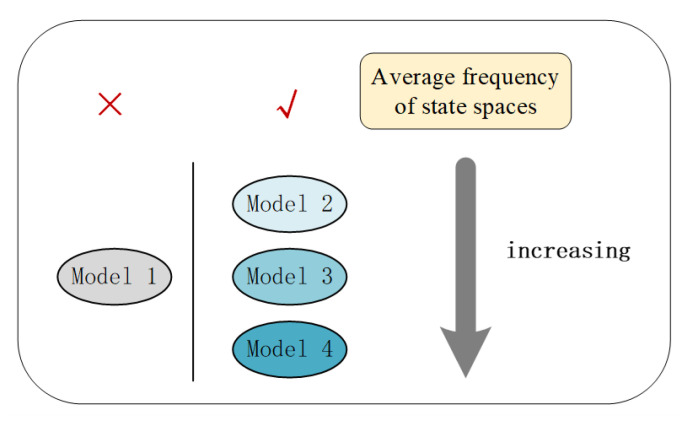
Comparison of the average of state spaces frequencies.

As shown in Figure 8, the average frequency of each state space in model 1 is 17.16. The average frequencies of each state space in models 2–4 are 4.27, 4.69, and 4.92, respectively. The average state spaces frequencies of four models are compared, which were shown in Figure 9.

As shown in Figure 9, model 1 only inputs vehicles in the directions of south and north. The distribution of state spaces is more concentrated and the average frequency is larger. Models 2–4 have a similar number of state space categories. As the balance of vehicle distribution becomes more comprehensive, the average frequency of each state space becomes larger. It indicates that the range of knowledge that the model can explore is becoming more complete.

### 4.3. Performance and Adaptation Analysis

**Figure 10:**
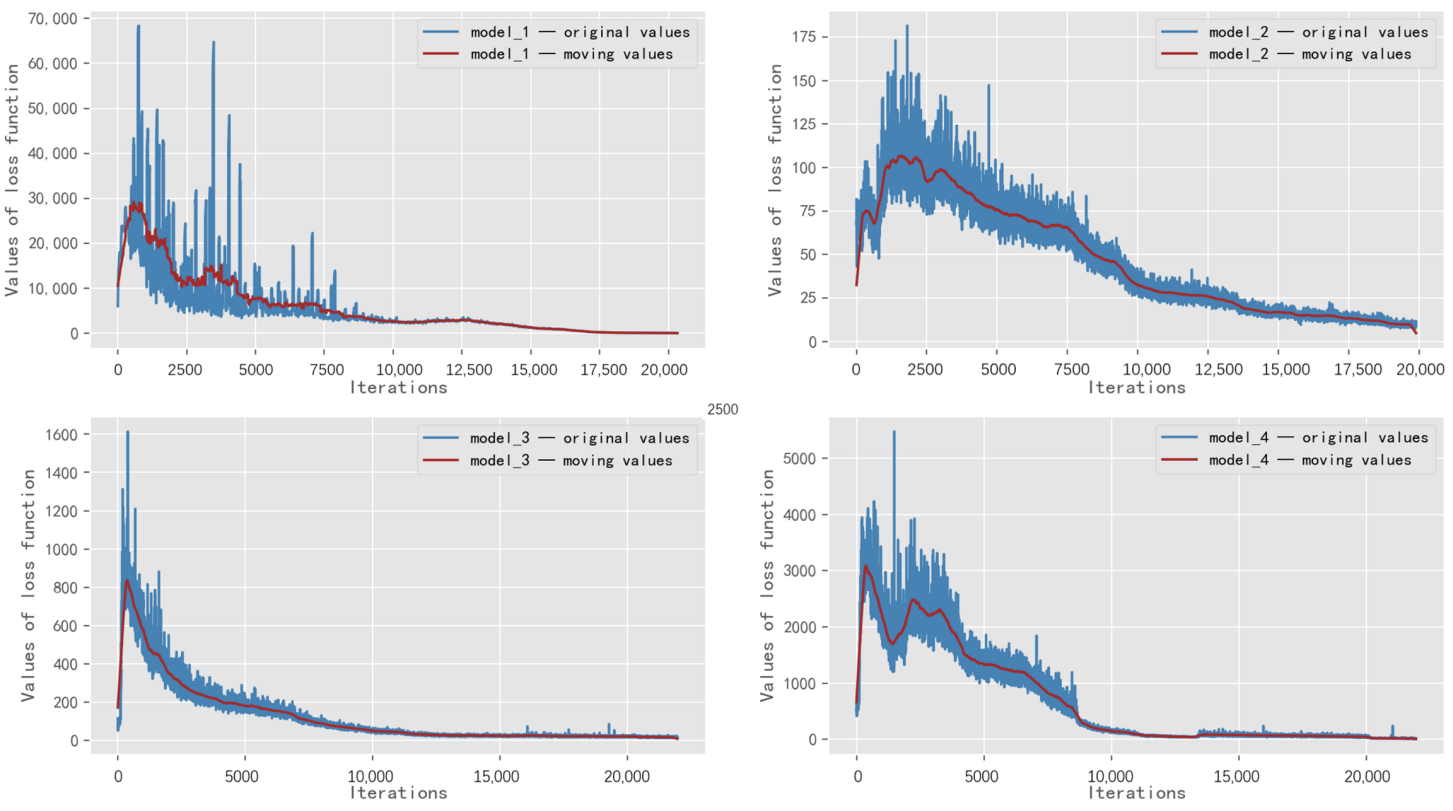
Variation of loss function for the four models.

The indicator of the loss function reflects how well the model is trained. The smaller the value of the loss function, the better the model is trained. Based on the above process for training, the variation of the loss function of each model is shown in Figure 10.

As shown in Figure 10, the four DQN models eventually converge. The loss function of Model 1 is of a larger order of magnitude, due to the extreme case of the traffic scenario. The loss functions of models 2–4 present a trend of gradually growing larger. The traffic scenario of Model 2 is the most balanced. The empirical knowledge used by the neural network for training is similar. Therefore, the fluctuation of the loss function is slight. While the traffic scenario of Model 4 is of different levels of balance, the empirical knowledge used by the neural network for training is complex and diverse. Therefore, the loss function fluctuates drastically.

The indicators of the average delay, loss time, and average cumulative delay are selected to evaluate the control performance of the DQN model. The average delay refers to the average delay time of all detected vehicles at a certain moment. The loss time refers to the cumulative loss time of all detected vehicles within a certain time interval.The average cumulative delay refers to the average cumulative delay time of all detected vehicles within a certain time interval.

Simulation experiments are conducted using the traffic scenarios during training, and the indicators of evaluation are presented in Table 8.

**Figure 11:**
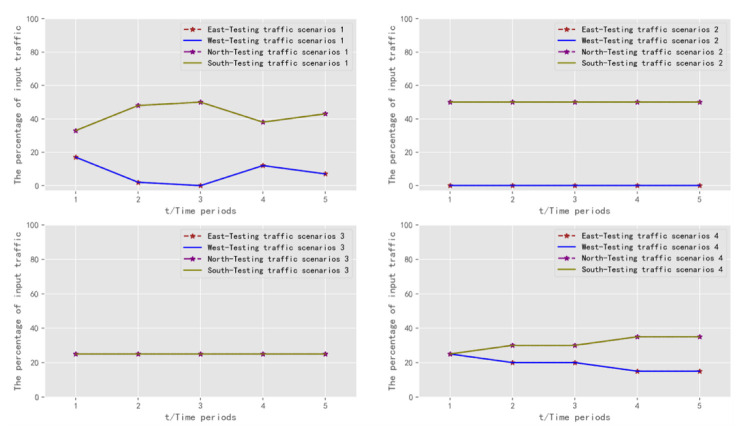
Diagram of traffic scenarios for testing: allocation of vehicles in each direction.

The result of experiments shows that the average delay, lost time, and cumulative average delay are at a stable and excellent level. It verifies the feasibility of the DQN algorithm for signal control. To further validate the adaptability of the model, four new traffic scenarios are designed for testing, which are shown in Table 9 and Figure 11.

The experiments are conducted using the new traffic scenarios, and the evaluation indicators are presented in Table 10, Table 11 and Table 12.

In the same test traffic scenario, comparing the average delays of all four models, Model 1 shows poor performance. It indicates that the knowledge learned by the model trained under extreme traffic scenarios is very limited and cannot be adapted to other traffic scenarios. Model 2 shows better performance in test traffic scenarios 1 and 4, but poorer performance in test traffic scenario 2. Model 3 and Model 4 are basically adaptable to all test traffic scenarios. However, the comparison reveals that Model 4 has better test results, indicating that it can achieve excellent control under other traffic demands as well. In the same test demand scenario, a comparison of lost time and average cumulative delay for the four models show a similar pattern to the average delay.

The results of the experiments show that Model 1 has poor levels of all evaluation indicators in each new test scenario compared to the other models. Therefore, it is less adaptable to new scenarios. Model 4 exhibits a better level of each evaluation metric in each new test scenario compared to the other models. Thus, it is more adaptable than the other models. The study shows that traffic scenarios should be designed to be comprehensive so that the number of state spaces is high, and the frequency of occurrence is also high. Once the model learns knowledge in as many comprehensive state spaces as possible, it would have the ability to adapt to new scenarios.

### 4.4. Execution Performance Comparison

**Figure 12:**
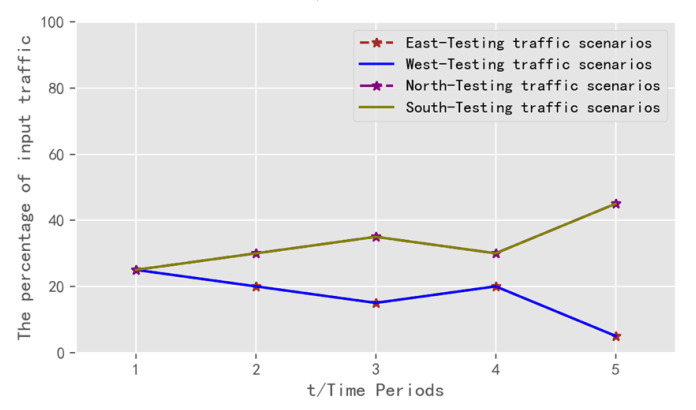
Diagram of traffic scenario 1 for testing: allocation of vehicles in each direction.

In this research, the DQN model with the best adaptation is compared with the SAC signal control model [30], the adaptive signal control method and the multi-time fixed timing method. Average cumulative delay and average delay are selected as the indicators for evaluation. Two traffic scenarios are designed for testing. The first traffic scenario for testing is designed as shown in Table 13 and Figure 12.

**Figure 13:**
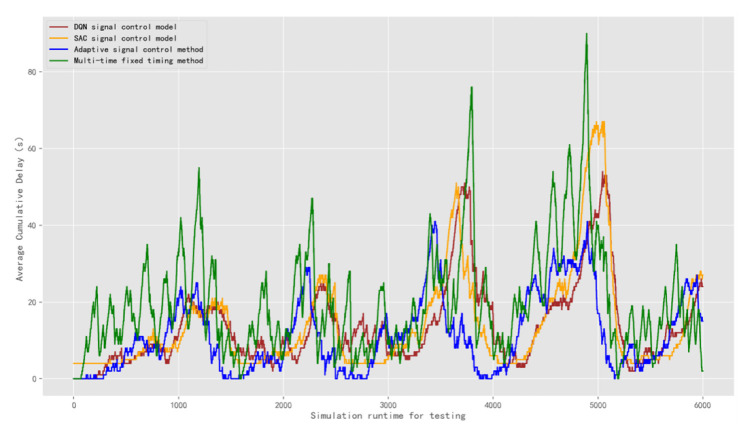
Comparison of the average cumulative delay of signal control methods under test traffic scenario 1.

The result of the average cumulative delay is obtained as shown in Figure 13. In the first test scenario, the average cumulative delay of the DQN model is 13.75 s. The average delay of the SAC model is 14.02 s. The average cumulative delay value of the adaptive control method model is 11.34 s and the multi-time fixed timing method is 19.87 s. The result shows that the adaptive signal control method achieve a better control effect for the signal control problem of an isolated intersection. The average cumulative delay data of the DQN model is a bit worse than it, but the gap is small. In addition, its control performance is better than that of the multi-time fixed timing method.

**Figure 14:**
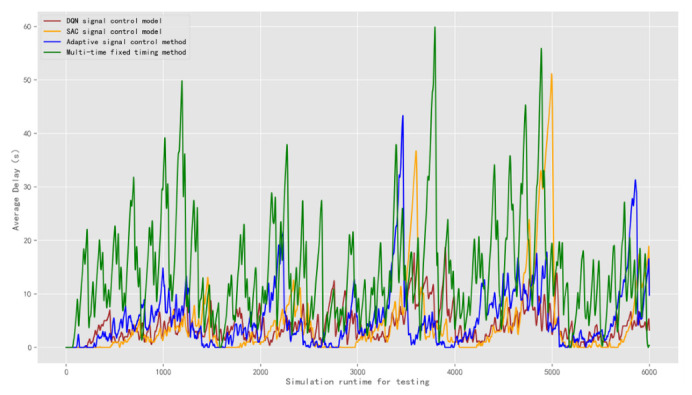
Comparison of the average delay of signal control methods under test traffic scenario 1.

The result of the average delay is obtained as shown in Figure 14. The average delay of the DQN model is 4.11 s. The average delay of the SAC model is 4.42 s. The average delays of the adaptive control model and the multi-time fixed timing method are 5.48 s and 14.15 s, respectively. Compared with the popular SAC signal control model, the average delay of the DQN model is reduced 8%. Compared with the adaptive signal control method, the average delay time is reduced 33%. The adaptation of the multi-time fixed timing signal control method in complex traffic scenarios is not ideal, indicating that it is unable to respond to diverse traffic demands and has some limitations.

In the first, second, and fourth periods, traffic demand with a more balanced traffic distribution state is provided. Our proposed model and the SAC model exhibit similar control performance in this case. In the third and fifth time periods, traffic demands with more extreme traffic distribution states are provided. Our proposed model exhibits more stable control performance, but the SAC model performs poorly and cannot adapt to the traffic scenario. Therefore, our model shows better performance than the advanced SAC model.

The second traffic scenario for testing is designed as shown in Table 14.

**Figure 15:**
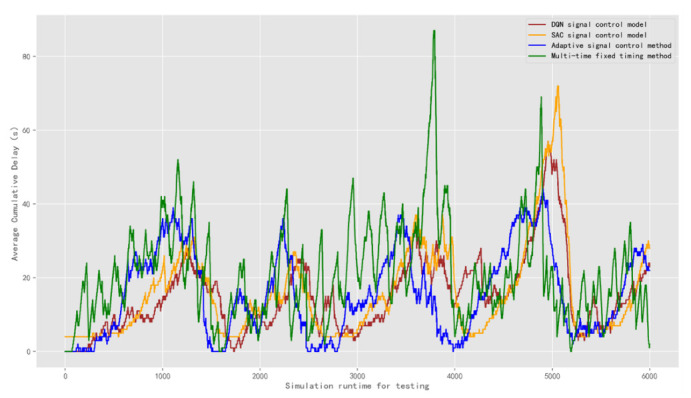
Comparison of the average cumulative delay of signal control methods under test traffic scenario 2.

The result of the average cumulative delay is obtained as shown in Figure 15. In the first test scenario, the average cumulative delay of the DQN model is 15.13 s. The average delay of the SAC model is 15.75 s. The average cumulative delay of the adaptive control method is 15.69 s and the multi-time fixed timing method is 20.99 s. The result shows that the performance of the DQN model is better than that of other methods.

**Figure 16:**
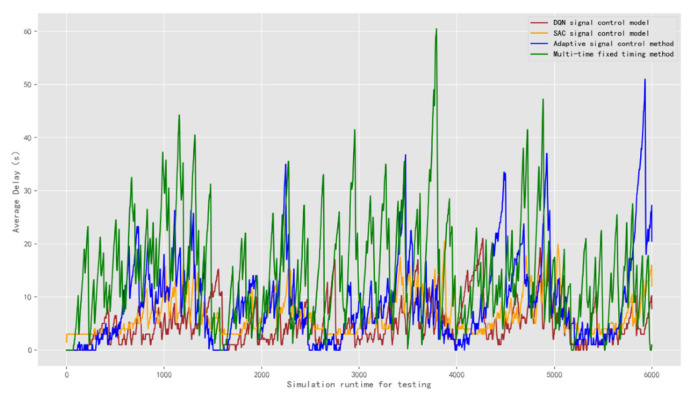
Comparison of the average delay of signal control methods under test traffic scenario 2.

The result of the average delay is obtained as shown in Figure 16. The average delay of the DQN model is 5.13 s. The average delay of the SAC model is 6.52 s. The average delays of the adaptive control model and the multi-time fixed timing method are 9.08 s and 14.78 s, respectively. Compared to the popular SAC and the adaptive signal control models, the average delay of the DQN model is reduced. The multi-time fixed timing signal control method cannot adapt to complex traffic scenarios and it has poorer performance.

In general, for the indicator of average cumulative delay, our proposed DQN model generally exhibits similar performance to the SAC signal control model and the adaptive signal control method. For the indicator of average delay, the DQN model exhibits the best signal control results. The result is related to the optimization objective we selected to train the DQN model. Since the reward function is set to minimize the average delay of the vehicles, the DQN model performs significantly better in signal control for the average delay.

## 5. Discussion and Conclusions

In this article, we provided a reasonable platform to test and compare various DRL algorithms for isolated traffic signal control. The main contents of this research are summarized as follows.

(1) To find a suitable model that could be applied to more than one intersection scenario, we designed four typical traffic scenarios as the input of the model to research. The designed traffic scenarios were extreme traffic states, perfectly balanced traffic states, perfectly balanced and mildly unbalanced traffic states, and fully balanced traffic states. It was found that the more complex the designed traffic demand, the more the traffic scenarios contained more traffic states, and the greater the possibility for the model to explore the knowledge fully. The result showed that the model with the traffic scenario of fully balanced traffic states had a better ability to adapt to new traffic scenarios.

(2) To verify the performance of our proposed signal control model, we compared it with other signal control methods, which included the multi-time fixed timing method, the adaptive control method, and the SAC model. The test results showed that our model achieved excellent performance. It worked better for signal control at an intersection than the popular SAC model.

(3) In real traffic systems, traffic demand has a high dimensionality. It takes much time and effort to train and calibrate the parameters for each intersection to obtain a signal control model. In this research, the training method with an adaptive performance model was provided by enriching the samples, which were used in learning. This enabled the model to be extended to more intersections and it provides a potential and feasible solution for urban signal control.

These test results are useful for many intelligent traffic control tasks, including how to use the DRL algorithm to solve complex traffic demand problems, including multi-intersection signal control. There is an autonomous driving demonstration area at Yizhuang, Beijing, where some of the advanced sensors are laid down. Our proposed model will be well integrated with real traffic systems for implementation purposes. For future work, we will investigate how to reasonably implement an adaptable DQN model to a real road network. Constrained by page limits, we will discuss these issues in the following articles.

## Figures and Tables

**Table 1:** Types of traffic data acquired by different sensors.

Technology	Loop Coil Sensor	Video, Radar Sensors	Connected Vehicles
Type of traffic data	*√*Traffic Volume	*√*Traffic Volume	*√*Traffic Volume
	*√*Vehicle Type	*√*Vehicle Type	*√*Vehicle Type
	×Occupancy	*√*Occupancy	*√*Occupancy
	×Speed	*√*Speed	*√*Speed
	×Vehicle Location	×Vehicle Location	*√*Vehicle Location
	×Trajectory	×Trajectory	*√*Trajectory

**Table 2:** Comparison of sensors, detailing the range of applications and advantages and disadvantages of different sensors.

	Loop Coil Sensor	Video, Radar Sensors	Connected Vehicles
Scope of detection	section detection	area detection	holographic detection
Advantage	detection area is limited	obtains traffic information of one segment	obtains the entire intersection’s information with high accuracy
Disadvantage	prone to break down	highly influenced by the environment	technology is not mature

**Table 3:** Classification and comparison of DRL signal control methods.

Classification	Author	Approach	Data Source	Achievement
**Value-based** **signal control** **approaches**	Kim D. [24]	DQN	Connected vehicles	improved travel efficiency of vehicle on the road network
	Rasheed F. [25]	MADQN	Video, Radar Sensors	reduce the total travel time of the vehicles
	Song Li [18]	DQN-TSC	Connected vehicles	improve both the training efficiency and model performance
**Policy-based** **signal control** **approach**	Rizzo et al. [28]	PG	Video, Radar Sensors	reduce average delay and improve effective capacity
**Actor-Critic based** **signal control** **approaches**	Feng Mao [30]	SAC	Connected vehicles	provide typical model settings in the traffic signal control problem
	Tianshu Chu [15]	A2C	Loop Coil Sensor	its optimality and robustness over other MARL algorithms
	C. Li [31]	PPO	Video, Radar Sensors	optimize the fairness criterion

**Table 4:** Hyperparameter setting of DQN.

Hyperparameter	Initialized Value
horizon	6000
episode	200
iteration	4
experience replay buffer	50,000
batch size	200
learning rate α	0.0003
discount factor γ	0.9
green duration $g_{t}$	6
yellow duration $y_{t}$	3

**Table 5:** Design of traffic scenarios for training: allocation of vehicles in each direction.

	Percentage	t=1	t=2	t=3	t=4	t=5
**Model 1**	$r_{1}\left(\%\right)$	0	0	0	0	0
	$r_{2}\left(\%\right)$	0	0	0	0	0
	$r_{3}\left(\%\right)$	50	50	50	50	50
	$r_{4}\left(\%\right)$	50	50	50	50	50
**Model 2**	$r_{1}\left(\%\right)$	25	25	25	25	25
	$r_{2}\left(\%\right)$	25	25	25	25	25
	$r_{3}\left(\%\right)$	25	25	25	25	25
	$r_{4}\left(\%\right)$	25	25	25	25	25
**Model 3**	$r_{1}\left(\%\right)$	25	20	20	15	15
	$r_{2}\left(\%\right)$	25	20	20	15	15
	$r_{3}\left(\%\right)$	25	30	30	35	35
	$r_{4}\left(\%\right)$	25	30	30	35	35
**Model 4**	$r_{1}\left(\%\right)$	25	20	15	10	5
	$r_{2}\left(\%\right)$	25	20	15	10	5
	$r_{3}\left(\%\right)$	25	30	35	40	45
	$r_{4}\left(\%\right)$	25	30	35	40	45

**Table 6:** Division of queue length interval.

	$q_{1}$	$q_{2}$	$q_{3}$	$q_{4}$
Interval of queue length	0m	100m	200m	750m

**Table 7:** Setting of unit queue length interval.

	$l_{1}$	$l_{2}$	$l_{3}$
Unit queue length interval	10m	25m	550m

**Table 8:** Experimental results of the four models in the same traffic scenario as during training.

	Model 1	Model 2	Model 3	Model 4
**Average delay**	1.21	6.20	7.54	6.14
**Loss time**	4684.34	8639.28	8134.24	7774.52
**Average** **cumulative delay**	5.79	19.73	20.21	17.48

**Table 9:** Design of traffic scenarios for testing: allocation of vehicles in each direction.

Traffic Scenario	Description	Percentage	t = 1	t = 2	t = 3	t = 4	t = 5
Traffic scenario 1 for testing	New traffic scenario	$r_{1}\left(\%\right)$	17	2	0	12	7
		$r_{2}\left(\%\right)$	17	2	0	12	7
		$r_{3}\left(\%\right)$	33	48	50	38	43
		$r_{4}\left(\%\right)$	33	48	50	38	43
Traffic scenario 2 for testing	Traffic scenario 1 for training	$r_{1}\left(\%\right)$	0	0	0	0	0
		$r_{2}\left(\%\right)$	0	0	0	0	0
		$r_{3}\left(\%\right)$	50	50	50	50	50
		$r_{4}\left(\%\right)$	50	50	50	50	50
Traffic scenario 3 for testing	Traffic scenario 2 for training	$r_{1}\left(\%\right)$	25	25	25	25	25
		$r_{2}\left(\%\right)$	25	25	25	25	25
		$r_{3}\left(\%\right)$	25	25	25	25	25
		$r_{4}\left(\%\right)$	25	25	25	25	25
Traffic scenario 4 for testing	Traffic scenario 3 for training	$r_{1}\left(\%\right)$	25	20	20	15	15
		$r_{2}\left(\%\right)$	25	20	20	15	15
		$r_{3}\left(\%\right)$	25	30	30	35	35
		$r_{4}\left(\%\right)$	25	30	30	35	35

**Table 10:** Average delay of the four models for each test traffic scenario.

	Model 1	Model 2	Model 3	Model 4
Traffic scenario 1 for testing	802.35	5.24	4.33	4.32
Traffic scenario 2 for testing	−	263.85	6.76	8.14
Traffic scenario 3 for testing	816.90	−	16.52	5.64
Traffic scenario 4 for testing	941.09	6.30	−	5.30

**Table 11:** Loss time of the four models for each test traffic scenario.

	Model 1	Model 2	Model 3	Model 4
Traffic scenario 1 for testing	333954.63	7985.18	8679.57	7676.60
Traffic scenario 2 for testing	−	311758.38	14986.62	13072.37
Traffic scenario 3 for testing	1092345.52	−	19482.85	7858.67
Traffic scenario 4 for testing	898408.75	9379.50	−	8184.29

**Table 12:** Average cumulative delay of the four models for each test traffic scenario.

	Model 1	Model 2	Model 3	Model 4
Traffic scenario 1 for testing	807.15	17.14	18.30	15.00
Traffic scenario 2 for testing	−	270.07	25.96	25.59
Traffic scenario 3 for testing	872.74	−	46.30	17.90
Traffic scenario 4 for testing	951.15	20.97	−	16.74

**Table 13:** Design of traffic scenario 1 for testing: allocation of vehicles in each direction.

	Percentage	t = 1	t = 2	t = 3	t = 4	t = 5
Traffic scenario for testing	$r_{1}\left(\%\right)$	25	20	15	20	5
	$r_{2}\left(\%\right)$	25	20	15	20	5
	$r_{3}\left(\%\right)$	25	30	35	30	45
	$r_{4}\left(\%\right)$	25	30	35	30	45

**Table 14:** Design of traffic scenario 2 for testing: allocation of vehicles in each direction.

	Percentage	t = 1	t = 2	t = 3	t = 4	t = 5
Traffic scenario 2 for testing	$r_{1}\left(\%\right)$	20	15	25	15	5
	$r_{2}\left(\%\right)$	20	15	25	15	5
	$r_{3}\left(\%\right)$	30	35	25	35	45
	$r_{4}\left(\%\right)$	30	35	25	35	45

## Data Availability

Not applicable.
